# Supplementary material for: Distinguishing imported cases from locally acquired cases within a geographically limited genomic sample of an infectious disease
Source: Bioinformatics. 2022 Nov 28;39(1):btac761. doi: 10.1093/bioinformatics/btac761 (PMC9805578; doi:10.1093/bioinformatics/btac761)
Supplement: btac761_Supplementary_Data [file btac761_supplementary_data.pdf]

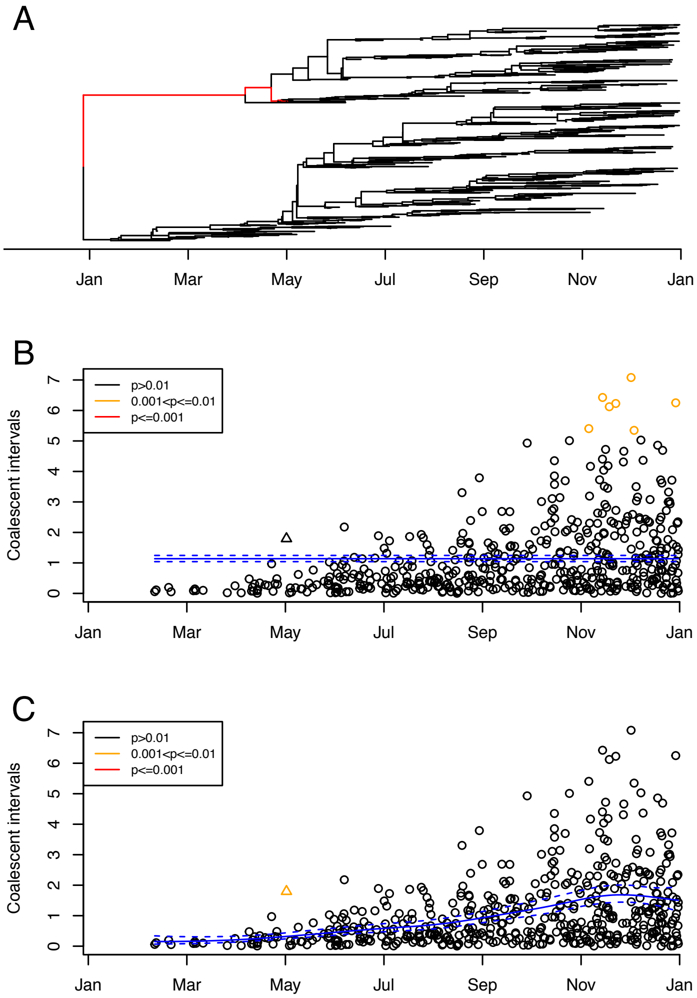

Figure S1: Illustrative application to a single simulated dataset showing that ignoring variations in the local population size can lead to false negatives in the detection of imports. A: Simulated phylogeny. B: Inference of imports under the model with constant population size. C: Inference of imports under the model with variable population size. In parts B and C, the inferred mean and 95% credible intervals of the mean coalescent intervals over time are shown in blue. The triangle in parts B and C corresponds to the first imported case shown in red in part A.

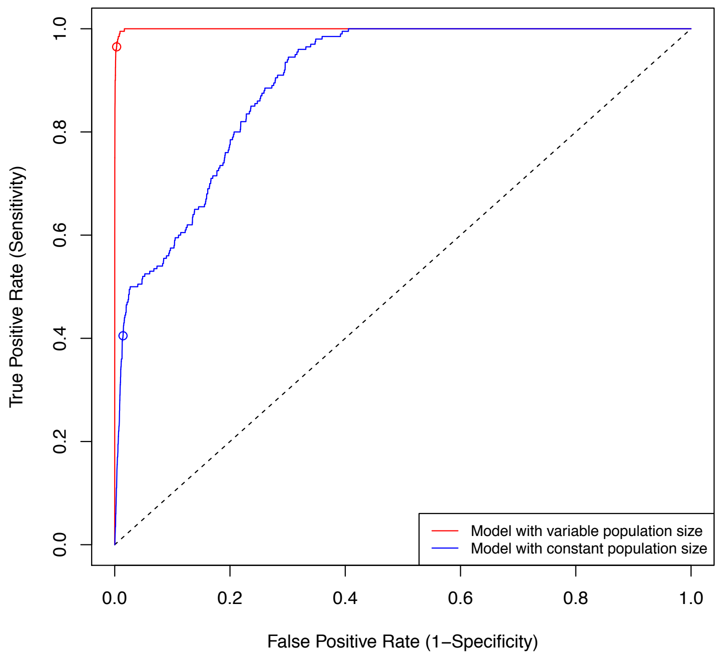

Figure S2: Receiver operating characteristic (ROC) curves for the model with variable population size (red) and the model with constant population size (blue). The dots represent a p-value of 0.01. Hyperparameter values $a_l = b_l = \sigma_\alpha = 2$ were used instead of $a_l = b_l = \sigma_\alpha = 5$ as in Figure 3.

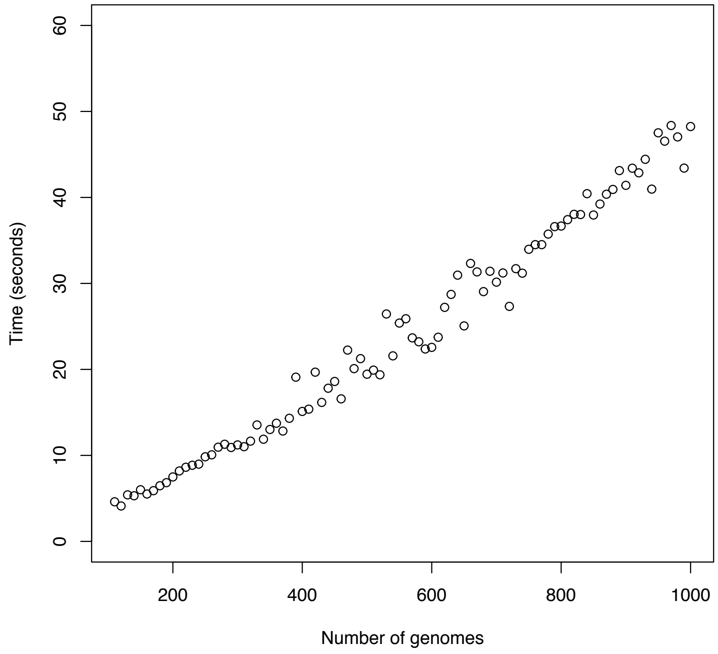

Figure S3: Wall time taken (y-axis) to run analyses in the default conditions under the variable population size model as a function of the number of genomes in the phylogeny (x-axis).

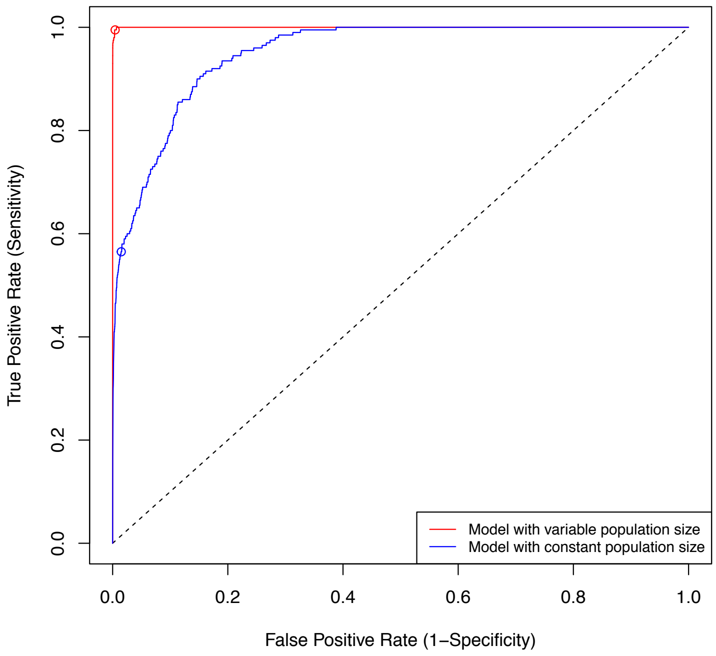

Figure S4: Receiver operating characteristic (ROC) curves for the model with variable population size (red) and the model with constant population size (blue). The dots represent a p-value of 0.01. A logistic growth model was used for the population size of each imported population instead of the linear growth model used in Figure 3.

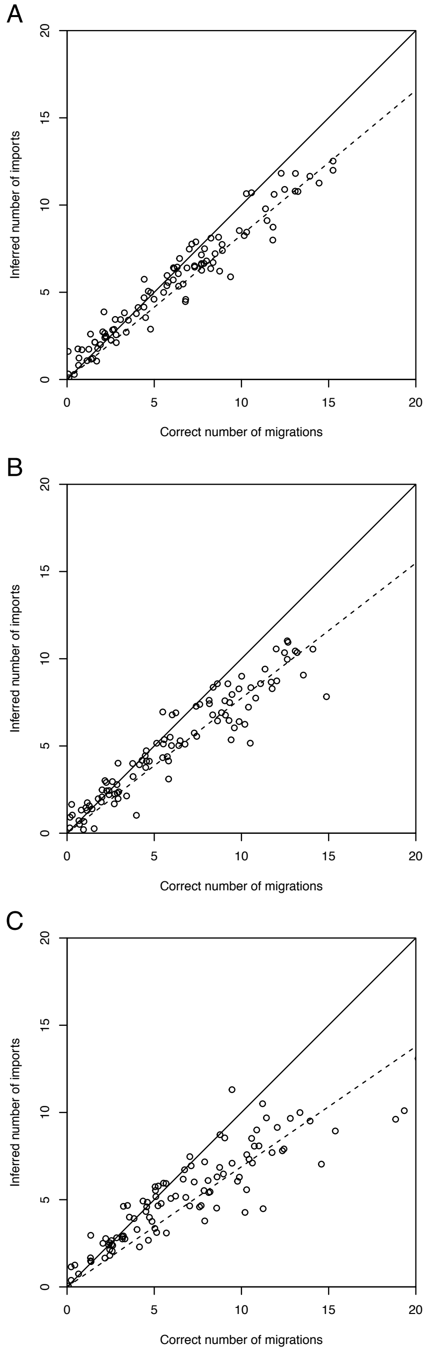

Figure S5: Same as Figure 4 but using the constant population size model instead of the variable population size model.

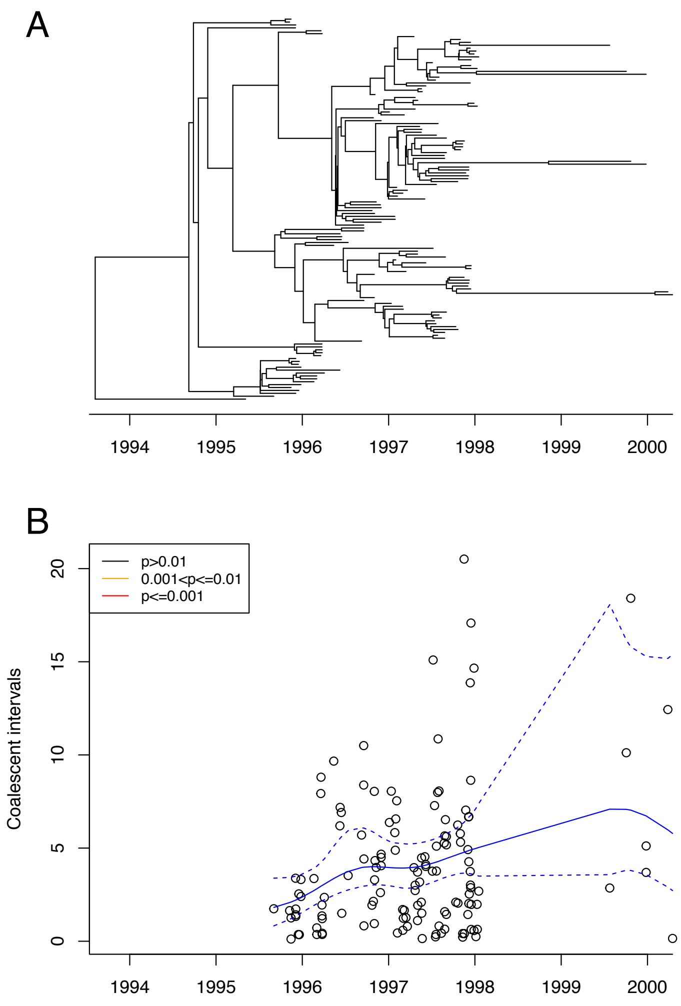

Figure S6: Application to an outbreak of gonorrhoea. A: Dated phylogeny. B: Inference of imports. The inferred mean and 95% credible intervals of the mean coalescent intervals over time are shown in blue.

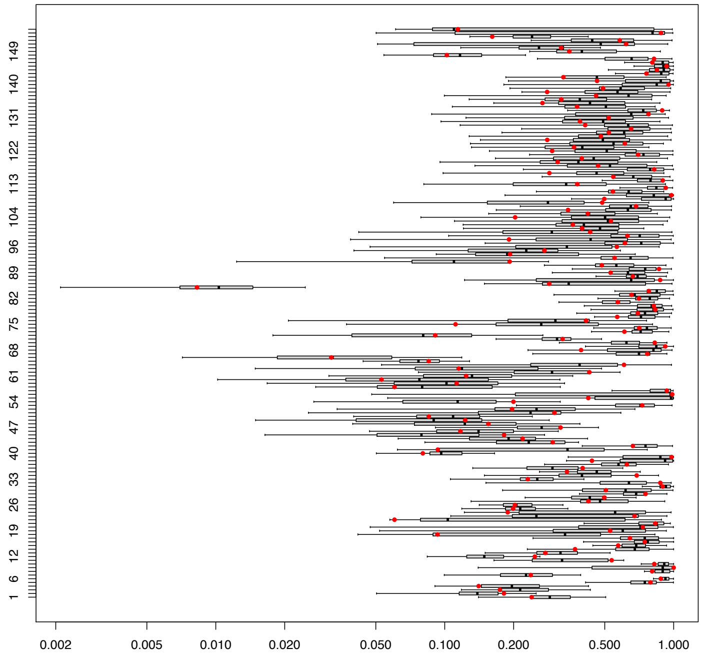

Figure S7: Application to *Shigella sonnei* dataset based on 100 dated phylogenies from the BactDating posterior sample. The boxplots show the distributions of p-value obtained for each case, and the red dots show the p-value computed from the consensus phylogeny.

Genome	Date	Lineage	Variant
EPI_ISL_532587	8/1/20	AD.2	
EPI_ISL_532620	8/2/20	B.1.1	
EPI_ISL_532118	8/4/20	B.1.389	
EPI_ISL_532024	8/5/20	B.1.235	
EPI_ISL_532105	8/7/20	B.1.1.279	
EPI_ISL_534413	8/10/20	B.1.1.372	
EPI_ISL_531473	8/11/20	B.1.1.311	
EPI_ISL_534647	8/16/20	B.1.1.303	
EPI_ISL_568421	8/16/20	B.1.258	
EPI_ISL_568423	8/18/20	B.1.441	
EPI_ISL_530768	8/21/20	B.1.221	
EPI_ISL_529685	8/22/20	B.1.282	
EPI_ISL_568393	8/22/20	B.1.416	
EPI_ISL_568293	8/25/20	B.1.1	
EPI_ISL_568360	8/26/20	B.1.1.277	
EPI_ISL_577371	9/2/20	B.1.1	
EPI_ISL_601940	9/17/20	B.1.1.255	
EPI_ISL_585313	9/19/20	B.1	
EPI_ISL_601175	9/22/20	B.1.1.307	
EPI_ISL_623930	10/22/20	B.1.1.170	
EPI_ISL_676414	11/4/20	B.1.1.7	Alpha
EPI_ISL_757190	11/18/20	B.1.1.317	
EPI_ISL_710633	12/4/20	B.1.36.26	
EPI_ISL_757231	12/7/20	B.1.36.29	
EPI_ISL_919311	12/12/20	B.1.177.17	
EPI_ISL_989986	12/16/20	B.1.351	Beta
EPI_ISL_997124	12/19/20	P.2	Zeta
EPI_ISL_865085	12/28/20	B.1.177	
EPI_ISL_1386678	2/6/21	B.1.562	
EPI_ISL_1247731	2/7/21	B.1.1.318	
EPI_ISL_1538076	3/31/21	B.1.525	Eta
EPI_ISL_1673240	4/11/21	B.1.1.7	Alpha
EPI_ISL_1741634	4/14/21	B.1.1.7	Alpha
EPI_ISL_1829569	4/23/21	B.1.617.2	Delta
EPI_ISL_2236724	5/17/21	B.1.617.2	Delta
EPI_ISL_2395821	5/24/21	B.1.617.2	Delta
EPI_ISL_2487803	6/2/21	B.1.1.7	Alpha
EPI_ISL_2700963	6/17/21	B.1.1.7	Alpha
EPI_ISL_3529997	8/4/21	P.1	Gamma
EPI_ISL_4600095	9/18/21	AY.34	Delta
EPI_ISL_7306019	11/28/21	AY.109	Delta
EPI_ISL_8763944	12/20/21	BA.1	Omicron
EPI_ISL_8197175	12/21/21	AY.120	Delta
EPI_ISL_8457600	12/26/21	AY.4	Delta
EPI_ISL_8457302	12/27/21	AY.98	Delta
EPI_ISL_8456419	12/28/21	AY.4	Delta
EPI_ISL_8440261	12/29/21	AY.4	Delta
EPI_ISL_9510674	1/27/22	BA.2.10	Omicron
EPI_ISL_10641104	2/23/22	XE	Omicron
EPI_ISL_11401173	3/16/22	BA.2	Omicron:1

Table S1: List of imports found in the SARS-CoV-2 dataset.
